# Characterization and Genetic Diversity of *Bacillus cereus* Strains Isolated from Baby Wipes

**DOI:** 10.3390/microorganisms10091779

**Published:** 2022-09-03

**Authors:** Laurenda Carter, Mei-Chiung J. Huang, Kyuyoung Han, Jayanthi Gangiredla, Jenny Yee, Hannah R. Chase, Flavia Negrete, Ben D. Tall

**Affiliations:** 1 Office of Applied Research and Safety Assessment, Center for Food Safety and Applied Nutrition, U.S. Food and Drug Administration, Laurel, MD 20708, USA; 2 Office of Cosmetics and Color, Center for Food Safety and Applied Nutrition, U.S. Food and Drug Administration, College Park, MD 20740, USA; 3 Office of Regulatory Affairs, San Francisco Laboratory, U.S. Food and Drug Administration, Alameda, CA 94502, USA

**Keywords:** *Bacillus cereus*, WGS, carbohydrate utilization, BTyper, genomic characterization, baby wipes

## Abstract

*Bacillus cereus*, a ubiquitous environmental microorganism known to cause foodborne illness, was isolated from samples taken from imported baby wipes from two different countries. These strains were characterized using a comprehensive molecular approach involving endpoint PCR, whole genome sequencing (WGS), comparative genomics, and biochemical analyses. A multiplex endpoint PCR assay was used to identify the enterotoxins: hemolysin BL, nonhemolytic enterotoxin, cytotoxin K, and enterotoxin FM toxin genes. Phylogenetically, the strains clustered into two major groups according to sequence type (ST) and singleton. We used the Center for Food Safety and Applied Nutrition (CFSAN) GalaxyTrakr BTyper computational tool to characterize the strains further. As an additional means of characterization, we investigated the possible role of carbohydrate transport systems and their role in nutrient uptake by performing a BLAST analysis of the 40 *B. cereus* genomes recovered from baby wipes. This study outlines a multifaceted workflow that uses the analysis of enterotoxigenic potential, bioinformatics, genomic diversity, genotype, phenotype, and carbohydrate utilization as a comprehensive strategy to characterize these *B. cereus* strains isolated from baby wipes and further our understanding of the phylogenetic relatedness of strains associated with baby wipe production facilities that could potentially pose an infection risk to a vulnerable infant population.

## 1. Introduction

During routine surveillance, the San Francisco U.S. Food and Drug Administration (FDA) lab identified 40 *Bacillus cereus* strains from samples taken from imported baby wipes. These wipes originated from two different manufacturing facilities in two different countries. *B. cereus* is a Gram-positive bacterium found in several categories of food products and is characterized as a widespread human pathogen associated with intoxication, diarrhea, and systemic clinical infections [1]. This spore-forming bacterium, known to cause a variety of diseases in humans [2], is ubiquitous in the natural environment and known to be pervasive in soil, dust, and eukaryotic plants. *B. cereus* displays a high diversity of lifestyles and ecological niches and includes beneficial as well as pathogenic strains. These strains are found on inert as well as on living surfaces, principally associated with biofilms [3]. Biofilms are complex communities that have also been mentioned to be a key element in the ability of *B. cereus* to colonize different environments and are suspected of playing a key role in the organism’s ubiquitous distribution and persistency [3]. The saprophytic life cycle of this organism has resulted in the contamination of irrigation water, vegetables, and many other foods [4].

Recognizing the ever-increasing prevalence and phylogenetic diversity found within the genus *Bacillus*, we advanced a previously described strategy used to characterize food-associated enterotoxigenic *B. cereus* strains with strains recovered from baby wipes. This approach, described by Carter et al. [5], detected the presence of hemolysin BL (hbl) *hblDAC*, nonhemolytic enterotoxin (nhe) *nheBAC*, cytotoxin K (*cytK*), and enterotoxin FM (*entFM*) toxin genes. In the current study, we used phylogenetic analyses, comparative genomics, several biochemical tests, and bioinformatics tools to characterize *B*. *cereus* strains recovered from baby wipes. This study aims to understand better the genomic diversity of *B. cereus* strains isolated from baby wipes and to increase the number of publicly available *B. cereus* genomes so that future phylogenomic studies can be performed.

## 2. Materials and Methods

### 2.1. Isolation of B. cereus Strains from Baby Wipes and Cosmetics Products

*B. cereus* strains were recovered from samples collected under a baby wipes surveillance sampling assignment conducted by the FDA. The FDA San Francisco Laboratory (SFL) used the Bacteriological Analytical Manual (BAM) protocols in Chapters 23 and 14 [6,7] to analyze the baby wipes samples. The samples collected from 10/2015 thru 11/2015 included two brands of wipes produced by two manufacturers in two separate countries (Table 1). Ten *B. cereus* strains were isolated from each brand. A total of 40 baby wipe strains were studied, along with nine strains obtained from cosmetics products such as liquid eyeliner and eye shadow samples. Nutrient agar slants inoculated with each isolate were received from SFL. Sterile loops were then used to inoculate the isolates from the slants onto BACARA chromogenic agar plates (bioMérieux, Hazelwood, MO, USA), followed by incubation overnight (o/n, 18–20 h) at 30 °C. Isolates with typical colony morphologies were confirmed as *B. cereus* by plating onto 5% sheep blood agar (TSAB, Remel, KS, USA) for demonstration of hemolytic activity. For quality control purposes, *B. cereus* type strain ATCC 14579 was used as a positive control for all plating and PCR experiments; *Bacillus thuringiensis* ATCC 29730 was used as a negative control to differentiate *B. cereus* from *B. thuringiensis*. Frozen stocks were maintained in tryptic soy broth (TSB) (BBL, Becton Dickinson, Franklin Lakes, NJ, USA) supplemented with 50% glycerol and stored at −80 °C.

### 2.2. Extraction of DNA from Bacterial Strains

Samples from frozen stocks were plated onto 5% sheep blood agar and grown o/n at 30 °C. Single colonies from these plates were added to 6 mL of TSB and incubated with shaking (180 rpm) at 30 °C o/n. Two mL aliquots of these cultures were pelleted (5000× *g* for 10 min., 4 °C) and the supernatants were discarded. Genomic DNA from each strain was isolated from the pellets using an UltraClean microbial DNA isolation kit (MO BIO Laboratories, Carlsbad, CA, USA) according to the manufacturer’s instructions. The DNA, eluted to concentrations of approximately 30–260 ng/μL, was recovered in certified DNA-free Tris buffer (10 mM Tris, pH 8) and stored at −20 °C until needed.

### 2.3. PCR Amplification of Enterotoxin Genes

Enterotoxin gene profiles were determined using an endpoint PCR assay with primers specific for *hblDAC*, *nheBAC*, *cytK*, and *entFM*. Primer sequences used were those described by Ngamwongsatit et al. [8] and Thaenthanee et al. [9] and PCR assay parameters as described by Carter et al. [5] (Supplemental Table S1). The amplification reactions were carried out in an Applied Biosystems 2720 Thermal cycler (AB, Applied Biosystems, Inc., Foster City, CA, USA).

Amplicons were separated in a 1.5% agarose gel using a 1 Kb DNA molecular size standard (Invitrogen, Carlsbad, CA, USA) to estimate amplicon sizes.

### 2.4. Whole Genome Sequencing (WGS) of B. cereus Strains from Baby Wipes and Cosmetics

A total of 49 *B. cereus* genomes were used for comparative genomic analyses. This included 40 genomes of *B. cereus* strains isolated from baby wipes samples and nine genomes from strains obtained from liquid eyeliner and eye shadow samples. Library preparations and WGS were completed for all strains using a Nextera XT Library Kit and MiSeq sequencer platform, respectively (Illumina, San Diego, CA, USA), as described by Carter et al. [5]. Raw sequence reads (FASTQ datasets) from Illumina sequencing were trimmed for removal of adaptor sequences and quality control purposes and de novo assembled using the CLC Genomics Workbench version 9.0 (CLC bio, Aarhus, Denmark). For annotation, FASTA files of the assemblies were uploaded onto the Rapid Annotation Subsystems Technology (RAST) server (online annotation; http://rast.theseed.org; accessed on 8 April 2020) [10], and for routine prokaryotic genome annotation using the Prokaryotic Genome Annotation Pipeline (PGAP) [11], at the National Center for Biotechnology Information (NCBI).

### 2.5. Comparative Genomics Analyses of B. cereus Strains Isolated from Baby Wipes

**Figure 1:**
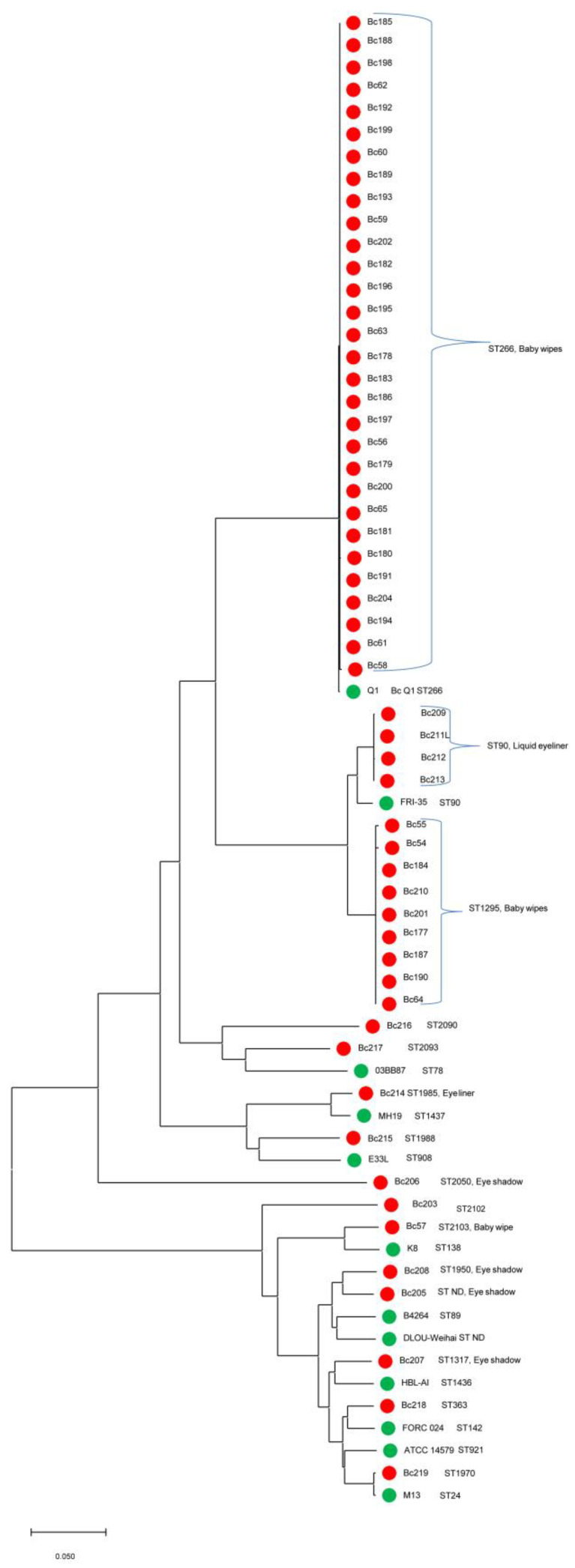
Phylogenetic and evolutionary history among 55 *B. cereus* strains isolated from baby wipes and other cosmetics products in comparison with NCBI reference genomes was inferred using the Neighbor-Joining method [15]. The optimal tree with the sum of branch length = 1.41269531 is shown. The tree is drawn to scale, with branch lengths in the same units as those of the evolutionary distances used to infer the phylogenetic tree. The evolutionary distances were computed using the p-distance method [16] and are in the units of the number of base differences per sequence. All ambiguous positions were removed for each sequence pair (pairwise deletion option). There was a total of 431,768 positions in the final dataset. Evolutionary analyses were conducted in MEGA X 10.0 [17]. The scale bar represents a 0.050 base substitution per site. The baby wipe *B. cereus* strains clustered into two distinct phylogenetically related groups according to ST. For example, the ST266 strains clustered with ST266 reference strain BcQ1 and the ST1295 strains clustered separately in a related but distinct clade with the ST90 strains, which grouped with the ST90 reference strain Bc FRI-35. The baby wipe and cosmetic product isolates are identified by red circles. The green circles represent the closest Bc reference strains.

The CFSAN BTyper tool (GalaxyTrakr Version 2.0.3), a *B*. *cereus* subtyping tool for characterization of *B. cereus* genomes for genomic signatures of virulence genes, sequence types (STs), and pantoate-beta-alanine ligase gene *panC* clade determinations, was used to characterize the *B. cereus* strains using FASTA files as input [12,13]. Using Single Nucleotide Polymorphism (SNP) analysis, a phylogenetic tree (Figure 1) was developed based on SNP profiles of 410 core genes from the baby wipes and cosmetics *B. cereus* strains [14].

### 2.6. Carbohydrate Utilization Studies

#### 2.6.1. Vitek^®^ 2 Compact System

Carbohydrate utilization measurements using the Vitek^®^ 2 Compact system (software version 5.0) (bioMérieux, Inc., Durham, NC, USA) were performed by SFL as part of the routine identification of the isolates.

Bionumbers were generated according to the utilization of each substrate contained within a VITEK 2 Bacillus Identification Card by an isolate from a baby wipe, liquid eyeliner, or eye shadow sample. The Vitek^®^ automation system uses a colorimetric reagent card (BCL) to identify spore-forming Gram-positive bacilli (i.e., *Bacillus* and related genera) (bioMérieux, Inc., Durham, NC, USA).

#### 2.6.2. API^®^ 50CH

API^®^ 50CH test strips (bioMérieux, Inc., Durham, NC, USA) combined with API^®^ 50 CHB/E medium [Ref 50 430 in kit brochure] for the identification of *Bacillus* and related genera were used for the determination of carbohydrate utilization. This kit is based on a growth-dependent test to determine the utilization of specific carbohydrates (bioMérieux, Inc., Durham, NC, USA). The kit was used according to the manufacturer’s instructions (bioMérieux, Inc., Durham, NC, USA). A cell suspension with a turbidity equivalent to a McFarland 2 standard or approximately 6 × 10^8^ CFU/mL was made in the medium with the *B. cereus* organisms after an initial overnight growth period at 30 °C on BACARA plates. The cell suspensions were made up of a pure culture of *B. cereus* after the isolates were grown on BACARA chromogenic agar plates (specific for the enumeration of *Bacillus cereus*), identified by the typical *B. cereus* colony morphologies, and plated on 5% sheep blood agar for confirmation.

The inoculation medium was API^®^ 50 CHB/E (tryptone 10 g/L, yeast extract 5 g/L, K_2_HPO_4_ 0.25 g/L, MnSO_4_ 0.05 g/L, and bromocresol purple 0.17 g/L). API^®^ 50CH samples were incubated for 2 days at 30 °C. Only one sugar was present in each well. If the sugar was fermented by the strain, a lowering of the pH caused a color change from purple to yellow.

### 2.7. Biochemical Carbohydrate Utilization Tests

A carbohydrate utilization assay was used to determine whether the *B. cereus* strains could utilize specific carbohydrates and produce acid and gas. We analyzed D-ribose, methyl α-D-glucopyranoside (unit of cyclodextrin), methyl β-D-glucopyroside (arbutin), cyclodextrin, and N-Acetyl-glucosamine. Phenol Red broth medium (Ramel, Lenexa, KS, USA) was supplemented with 1.8 g of each individual carbohydrate to a final concentration of 1%. The broth solution was then sterilized using a Nalgene filter unit (Nalgene Nunc International, Rochester, NY, USA) pore size of 0.22 µm. An inverted Durham tube 6 × 50 mm (Thomas Scientific, Swedesboro, NJ, USA) was added to a 16 × 100 mm tube to capture gas production. These tubes were then sterilized by autoclaving. Six mL of the filter-sterilized carbohydrate medium was subsequently added to each tube. Two to three *B. cereus* colonies were added to a 12 × 75 mm tube containing 3 mL of saline to achieve an OD_600_ reading of 0.3, which equals ~10^8^ CFU/mL. A 100 µL aliquot of this cell suspension was then added to the culture tubes containing the phenol red broth, sugar, and Durham tube and incubated at 30 °C. The tubes were monitored for seven consecutive days to observe phenotypic results (red to yellow color change in the media).

## 3. Results

### 3.1. Prevalence and Distribution of B. cereus Enterotoxin Genes in Baby Wipes Strains

All the strains were hemolytic and possessed lecithinase activity and were PCR-negative for the *B. thuringiensis* insecticidal crystal toxin, *cry* gene, as expected. The prevalence and distribution of enterotoxin genes such as hemolysin BL(hbl) *hblDAC*, nonhemolytic enterotoxin (nhe) *nheBAC*, cytotoxin K (*cytK*), and enterotoxin FM (*entFM*) genes found in the 40-baby wipe strains are shown in (Table 2). Six different enterotoxin gene profiles among the strains were identified. Profile 1 consisted of strains that were PCR-positive for: *nheB*, *cytK*, and *entFM*. Strains PCR-positive for *nheB* and *entFM* were grouped into profile 2. All four strains identified in profile 3 were PCR-positive for all toxin genes except *cytK*. Seven strains were included in profile 4. The most prevalent enterotoxigenic gene profile, profile 5, was detected in 20 strains; these strains were PCR-positive for *nheBAC* and *entFM* genes. The difference between profiles 4 and 5 was the presence of *cytK* in profile 4. The one strain in profile 6 was PCR-positive for *nheBC*, *cytK*, and *entFM*. Thirty-one strains out of forty were PCR-positive for *nheBAC* genes. All strains were positive for *nheB* and *entFM*. The presence of all the *hbl* genes (*hblDAC*) was found in only four strains. Interesting to note that seven of nine ST1295 strains were categorized under profile 4. From our previous characterization studies of *B. cereus* strains that were associated with contamination of foods, the hemolysin *hbl* was the most prevalent toxin gene observed [5].

We compared our PCR analysis results to the BTyper CFSAN GalaxyTrakr BTyper computational tool. The results obtained from the PCR analysis matched 100% for the presence of *entFM* and *nheB* virulence genes as determined by the BTyper analysis. With the BTyper computational tool, there was a 100% match for *nheABC* genes but only a 31% agreement with *nheA* alone. There were 11 strains that were identified as positive for the presence of the *cytK* toxin gene in the PCR assay, but only 10 were found to be positive with the BTyper tool.

Isolates from Manufacturer A, Europe (pure baby wipes) had toxin genes with profile #s 1, 2, and 3. Manufacturer A, from Europe, gentle baby wipes had toxin gene profiles that were predominately under profile #5, with a few exceptions falling under profile #4. The manufacturer of scented baby wipes, Manufacturer B, from Asia, also had a majority of isolates falling under profile #5, two under profile #4 and one under profile #3. Isolates from Manufacturer A from Europe were found to have toxin profiles that were decidedly different not only from the other manufacturers of baby wipes and other countries but also from the same manufacturer in the same country, differing only by the particular brand, which was the pure baby wipes. Whether a particular manufacturer and or the brand being produced is responsible for the differences observed in the toxin gene profile is a question that remains to be answered.

### 3.2. Whole Genome Sequencing (WGS) of B. cereus Strains from Baby Wipes and Cosmetics

A total of 49 *B*. *cereus* genomes were used for comparative genomic analyses. This included 40 genomes of *B. cereus* strains isolated from baby wipe samples and nine genomes from strains obtained from liquid eyeliner and eye shadow samples. Library preparations and WGS were completed for all strains using a Nextera XT Library Kit and MiSeq sequencer platform, respectively (Illumina, San Diego, CA, USA), as described by Carter et al. [5]. Raw sequence reads (FASTQ datasets) from Illumina sequencing were trimmed for removal of adaptor sequences and quality control purposes and de novo assembled using the CLC Genomics Workbench version 9.0 (CLC bio, Aarhus, Denmark). For annotation, FASTA files of the assemblies were uploaded onto the Rapid Annotation Subsystems Technology (RAST) server (online annotation; http://rast.theseed.org; accessed on 13 July 2022) [10], and for routine prokaryotic genome annotation using the Prokaryotic Genome Annotation Pipeline (PGAP) [11], at NCBI. WGS assembly and genomic characterization of *B. cereus* strains recovered from baby wipes are shown in (Table 3). Nucleotide sequences of these strains were deposited into NCBI’s GenBank and released to the public by submission to NCBI under *B. cereus* GenomeTrakr Project, CFSAN BioProject number PRJNA326742, which is part of the CFSAN umbrella foodborne pathogen research Bioproject (PRJNA186875).

### 3.3. Phylogenetic Analysis/Comparative Genomics

Using Single Nucleotide Polymorphism (SNP) analysis, a phylogenetic tree (Figure 1) was developed based on SNP profiles of 410 core genes from the baby wipes and cosmetics *B. cereus* strains. [14]. The tree showed that the baby wipes *B. cereus* genomes clustered into phylogenetically related groups according to ST. Two major clusters and a singleton were found and involved strains possessing ST266 and ST1295 and an ST2103 strain. The phylogenetically related isolates from cosmetics sources, i.e., liquid eyeliners and eye shadows, along with *B. cereus* reference genomes, were also added to the tree for comparison. Additionally, several cosmetics samples of unspecified origin were included in the tree. The detection of *B. cereus* in baby wipes and cosmetics products occurred concomitantly using surveillance samples under CFSAN’s Cosmetics Program. Therefore, we decided to include all these strains in the phylogenetic analysis to compare phylogenetic relatedness among the strains. The baby wipe strains, however, were only included in WGS analyses. Manually overlaying each strain (cluster) with ST data obtained from BTyper and PubMLST analyses showed that the baby wipes strains in each cluster grouped according to ST. The largest cluster, shown in Figure 1, revealed strains that were identified as ST266. Another cluster of baby wipe strains possessing the ST1295 designation was also grouped near a smaller cluster of four ST90 *B. cereus* strains isolated from liquid eyeliner samples along with one *B. cereus* reference strain. Moreover, MLST analysis showed that there are two allelic profile differences between ST90 and ST1295 (ST90: *ilv*: 41 versus 270 and *pyc*: 46 versus 6) strains, respectively.

**Figure 2:**
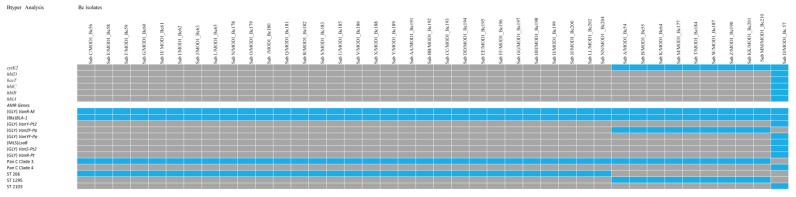
The heatmap represents the presence/absence of *B. cereus* virulence, antimicrobial resistance genes (AMR), *panC* phylogenic typing genes, and ST designations identified in baby wipe strains using BTyper. All the baby wipes strains possessed *nheBAC*, *entAFM*, *bpsEFH*, *cerAB*, *clo*, *plcABR*, and *inhA1A2*. ST1295 strains possessed the additional virulence factor, *cytK2*. The lone ST2103 strain carried *cytK2*, *hblDAC*, *bceT,* and *hblB* as additional virulence factors. There were seven AMR genes which included (GLY) *VanY-Pt2*, (GLY) *VanYF-Pp*, (MLS)*LsaB*, (GLY) *VanS-Pt2,* and (GLY) *VanR-Pt*, along with (GLY) *VanR-M* and (Bla) *BLA-1* found in all the strains. All strains except for Bc57 ST2103 were identified as *panC*-positive. Bc57 ST2013 was identified as a *panD*-positive strain. The presence of the gene is indicated in blue, and the absence is indicated in gray. The measured parameters were consistent with the ST of the strains.

CFSAN’s GalaxyTrakr BTyper computational tool identified virulence genes associated with each baby wipe strain, panC Clade typing, and antimicrobial resistance (AMR) genes shown in Figure 2 along with their ST designations. This tool demonstrated that all strains possessed *nheBAC*, *entAFM*, *bpsEFH*, *cerAB*, *clo*, *plcABR*, and *inhA1A2*. ST1295 strains possessed an additional virulence factor, *cytK2*. The ST2103 strain carried *cytK2*, *hblDABC*, and *bceT* as additional virulence factors. All but one strain was found to have the *panC* Clade 3 determinants. ST2103, Bc57 was identified as possessing a *panC* Clade 4 determinant.

The AMR genes found in ST266 strains were (Gly) *VanR-M* and *Bla_-1_*. ST1295 strains had the same AMR profile with one additional gene, (Gly) *VanZF-Pp*. ST2103 strain Bc57 possessed several AMR genes, including (Gly) *VanR-M*, *Bla_-1_*, (Gly) *VanY-Pt2*, (Gly) *VanYF-Pp*, (MLS) *LsaB*, (Gly) *VanS-Pt2*, and (Gly) *VanR-Pt* and grouped under *panC* Clade 4 determinant. *VanR* genes are involved in antibiotic resistance to glycopeptides. *Bla* and *MLS* genes are β-lactamase and macrolide-lincosamide-streptogramin resistance genes, respectively.

There were no plasmids present in these genomes (https://bio.tools/PlasmidFinder, last accessed on 19 August 2022). All strains were fosfomycin resistant except for Bc57, which shows resistance to doxycycline and tetracycline.

### 3.4. Annotation of Loci Involved in Carbohydrate Metabolism in the Strains Used in the Current Study

Four hundred and ten genes encoding for proteins involved in carbohydrate metabolism in the reference strain B. cereus ATCC 14579 were obtained from the ‘SEED’ viewer server at (https://rast.theseed.org; last accessed on 13 July 2022). A local database containing 40 genome assemblies from this study, along with the reference genome, was formatted for BLAST analysis following the instructions from the NCBI Blast suite. An 80% cutoff parameter was used to differentiate protein homology across different clusters. Data was generated based on the presence/absence of these shared homologous loci. Around 300 unique loci were included in this analysis and were used to correlate strain phenotypic carbohydrate utilization profiles with genotype.

### 3.5. Carbohydrate Utilization Studies

Three different technologies, Vitek^®^ 2 Compact protocol, API^®^ 50CH, and biochemical analyses, were used to identify carbohydrates utilized by the strains. All three tests demonstrated the ability to utilize several common carbohydrates.

**Figure 3:**
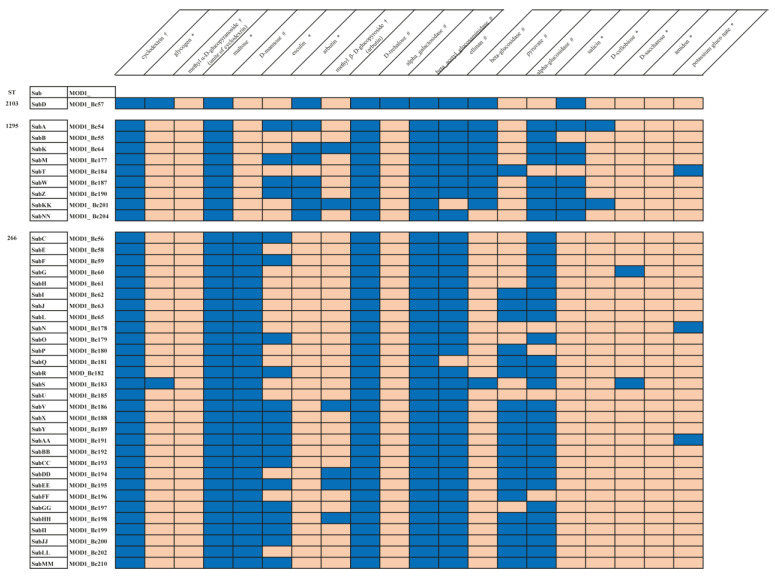
Comparison of carbohydrate utilization capabilities of the 40 *B. cereus* isolates from baby wipes.

The results of the API^®^ 50CH test showed that the strains were able to utilize the following carbohydrates: D-fructose, maltose, D-mannose, D-trehalose, D-glucose, N-acetyl D-glucosamine, and ribose. A biochemical carbohydrate fermentation test was used to manually test several carbohydrate substrates, which included: D-ribose, methyl α-D-glucopyranoside (unit of cyclodextrin), methyl β-D-glucopyroside (arbutin), cyclodextrin, and N-acetyl-glucosamine. All baby wipe strains could utilize D-ribose and N-acetyl-D-glucosamine within 24 h. In contrast, cyclodextrin utilization occurred between four and five days for most of the strains. Strains Bc57 and Bc179 needed 7 days to utilize cyclodextrin completely. Methyl β-D-glucopyroside (arbutin) was used in only six strains and developed within 96 h (Supplemental Table S2). The capacity to use methyl β-D-glucopyroside (arbutin) occurred within 48 h for Bc186. For Bc64, Bc195, Bc198, and Bc201, methyl β-D-glucopyroside (arbutin) was used within a 72-h period. Ninety-six hours of incubation was needed for methyl β-D-glucopyroside (arbutin) utilization to occur in strain Bc194. Methyl α-D-glucopyranoside (an isomer of cyclodextrin) was the only carbohydrate not used by any of the strains. The results comparing all three carbohydrate utilization studies are shown in (Figure 3). Not all the same carbohydrates were included in each of the three different carbohydrate utilization protocols. N-acetyl-glucosamine, D-glucose, trehalose, maltose, and maltotriose are compounds known to support the growth of *B. cereus* [18]. These carbohydrates had nearly identical positive results regardless of the test protocol used. A comparison of the different carbohydrate tests also revealed a different utilization capacity for the same carbohydrate.

The heatmap is based on three different carbohydrate testing protocols, VITEK (#), API^®^ 50CH (*), and Biochemical Fermentation tests (†) (BF). Growth or utilization is shown in blue, and no growth or utilization is depicted in orange. The three different STs are illustrated along with the identification of the isolates. Not all the same carbohydrates were included in each of the three different carbohydrate test panels. (i) Substrates N-acetyl-glucosamine and D-ribose were included in all three testing protocols, and D-glucose was included in two of the protocols. All the isolates were able to utilize these carbohydrates; (ii) carbohydrates with similar results, either positive or negative (with the exception of one or two isolates) using the different protocols, included cyclodextrin (#, †), glycogen (#, *), maltotriose (#), maltose (*), and D-trehalose (#, *); (iii) α -galactosidase (#) and amidon (*) only appear to be utilized by ST2103, Bc57; (iv) the carbohydrates appearing in the VITEK (#) protocol and were negative for substrate utilization in all the isolates included inulin, β-galactosidase, D-galactose, myo-inositol, methyl-D-xyloside, α -mannosidase, D-mannitol, D-melezitose, L-rhamnose, β-xylosidase, and palatinose. β-mannosidase and D-tagatose; (v) the carbohydrates in the API^®^ 50CH protocol (*), negative for utilization in all the isolates, included glycerol and D-lactose; (vi) esculin (*) and esculin hydrolysis (#) gave conflicting results. There were 20 isolates that were positive for utilization of esculin (*) and 12 isolates able to utilize esculin hydrolysis (#). ST1295 had four isolates positive for both esculin (*) and esculin hydrolysis, while ST266 only had one isolate positive for utilization of both carbohydrates; (vii) methyl-α-D-glucopyranoside acidification (#) and methyl α-D-glucopyroside (unite of cyclodextrin) (†) were the only two carbohydrate protocols tested that showed completely opposite results for utilization capabilities.

**Figure 4:**
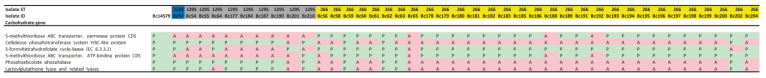
The presence/absence of genes related to carbohydrate utilization shows the closest agreement to ST found in all the strains showing differences in gene composition. P = presence of a gene; A = absence of a gene; Bc14579 is *B. cereus* ATCC 14579; ST57 is highlighted in light blue, ST1295 is highlighted in gray, ST266 is highlighted in yellow.

There were only six carbohydrate utilization-related genes that demonstrated a correlation between the presence/absence of genes and ST (Figure 4). These included the 5-methylthioribose ABC transporter permease protein gene, which was absent in ST2103 Bc57 and all ST1295 strains and present in all ST266 strains except for Bc65, Bc188, and Bc192. In contrast, the cellobiose phosphotransferase system YdjC-like protein gene was present in ST2103 Bc57 and all ST1295 except Bc201 but only present in three of the thirty ST266 strains, Bc59, Bc62, and Bc202. A 5-formyltetrahydrofolate cyclo-ligase gene (EC_6.3.3.2) was present in ST2103 Bc57, two ST1295 strains, and 28 out of a total of thirty ST266 strains. The 5-methylthioribose ABC transporter, ATP binding protein gene, was absent from the strain Bc57, ST2103, and from all strains, except Bc210, within the ST1295 cluster; the transporter was present in all but four strains within the ST266 cluster. Likewise, with respect to genes encoding the carbohydrate phosphoglycolate phosphatase and lactoylglutathione lyase and related lyases, a parallel could be drawn between presence/absence patterns and ST designations.

Pearson correlation was used to measure the linear correlation between the pattern of presence and absence of each pair of genes. The output value is between −1 and 1, where values near −1 show a strong negative correlation, 0 is no correlation, and values near 1 show a strong positive correlation. The formula uses numbers as input, so presence was assigned a value of 1, and absence was assigned a value of 0. Statistical analysis of the Pearson correlation coefficient (Supplemental Table S3) shows that A and D are highly correlated (5-methylthioribose_ABC transporter, permease and 5-methylthioribose ABC transporter, ATP-binding). Moreover, B, E, and F are highly correlated Cellobiose phosphotransferase system YdjC-like protein, Phosphoglycolate phosphatase, Lactoylglutathione lyase_and related_lyases).

The biochemical carbohydrate utilization assay was used to determine whether the *B. cereus* strains could utilize and produce acid and gas. The specific carbohydrates tested were, D-ribose, methyl α-D-glucopyranoside (unit of cyclodextrin), methyl β-D-glucopyroside (arbutin), cyclodextrin, and N-acetyl-glucosamine. Gas production occurred in all the sample tubes.

### 3.6. Genome Annotation Using RAST (Rapid Annotation Using Subsystem Technology)

Our investigation uncovered approximately 34 subsystems (equivalent to discrete pathways) that were involved in carbohydrate utilization in the baby wipe strains using *B. cereus* ATCC 14579 as the reference strain. More than 410 genes were annotated and determined to be involved in carbohydrate utilization. Two hundred ninety-seven unique genes from this set were used to query a local BLAST database. Genetic features encoding carbohydrate presence differed between the 40 baby wipe strains and within the two major STs, ST266 and ST1295, and the one singleton ST2103.

The carbohydrate utilization genes resulting from the BLAST analysis revealed a total of 99 genes with non-redundant presence/absence profiles among the strains (Supplemental Table S4). Only 22 of these carbohydrate utilization-related genes were present in ST266 Bc65. Ninety-three of these genes related to carbohydrate utilization were present in ST2103 Bc57. However, there were 16 carbohydrate utilization-related genes present in ST2103 Bc57 that were either present or absent in an inconsistent manner among all the other strains and STs. These 16 genes related to carbohydrate utilization, their function, and enzyme pathways (Table 4) are characterized using a combination of sequence databases (NCBI-BLAST), enzyme databases [19], and pathway databases [20]. Variation in the presence/absence of genes related to carbohydrate utilization between different STs was observed in most of the strains. A few examples of this include the gluconate transporter family protein gene, present in the ST2103 strain Bc57, all the ST1295 strains except Bc201, and only present in ST266 strains Bc59, Bc62, Bc63, and Bc202. Likewise, the glycerophosphoryl diester phosphodiesterase (EC3.1.4.46) gene (periplasmically secreted in Gram Positives) was absent in Bc64 and Bc201 within ST1295 strains and absent in all ST266 except for Bc 59, Bc62, Bc63, and Bc202. The results of the presence/absence of genes in all the strains are shown in (Supplemental Table S4).

## 4. Discussion

*Bacillus cereus*, known to be ubiquitous across many environmental niches, possesses pathogenic traits that have not only been found in foods but in cosmetics as well. *B. cereus* is a microbial contaminant that could adversely affect the product safety of cosmetics and facial toiletries and pose a threat to the user if other key risk factors are also present [21]. There have also been several studies over the years that have confirmed the impact of *B. cereus* commonality affecting vulnerable infant populations and neonates [22,23]. A recent study outlining *B. cereus* infection in neonates reported a suspected source of infection coming from packs of diapers and linen [24]. The potential pathogenicity that this organism may cause, found in these baby wipes, raises the question of what commonalities might exist that can be used to characterize these strains from two different manufacturers and to different geographically distant facilities.

*B. cereus* has been predominantly associated with foodborne illnesses [25]. Routine analysis of food samples for the presence of *B. cereus* is part of the food safety surveillance program at the FDA. As the result of a sampling assignment from the Office of Cosmetics and Colors (OCAC) at FDA/CFSAN, the SFL discovered the presence of this organism in imported baby wipes. The sources of this contamination are yet unknown. Even though Good Manufacturing Practices [26] were followed, contamination of end products may have occurred at any time during the manufacturing process, including raw materials, from the processing environment and the manufacturing process itself. There have been reports implicating *B. cereus* contamination in hospitals citing environmental reservoirs, including air filtration/ventilation equipment and linens [1]. *Bacillus* spores have been implicated in the contamination of raw materials, synthetic materials, and dry powders coming into manufacturing facilities [27].

Stewart et al. reported key microbial contaminants of personal care products (PCP). They described *Bacillus cereus* as ubiquitous in nature (soil, dust, cereal crops, plants, animal hair, fresh water, and sediments) and occurring naturally in a wide range of raw materials and foodstuffs. They describe its occurrence in PCPs as likely due to its presence in raw materials used in manufacture. [28]. Microorganisms are known to survive and grow in many different environments, sometimes under extreme growth conditions. Physiological responses to environmental change may include cellular metabolism and mechanisms of adaptation and resistance [4].

Whole genome sequencing, which has become a routine technique for food safety surveillance, was used for the analysis of these strains recovered from baby wipes. Genomic data can now be extended to the identification of microorganisms found in numerous different habitats. A comprehensive approach involving phenotypic and genotypic analyses was undertaken to establish a platform to characterize *B. cereus* strains recovered from baby wipes.

In this study, we generated a phylogenetic tree based on SNP profiles of 410 core genes from 40 baby wipes *B. cereus* strains and compared these genes to 61 whole genome sequences downloaded from NCBI. The strains clustered into two major groups according to sequence type (ST), ST266 and ST1295, and a singleton, ST2103. Most of the strains belonged to ST266. The closest reference strain, BcQ1, was isolated from a deep-subsurface oil reservoir in the Daqing oil field in northeastern China. This strain is described as being able to produce biosurfactants and survive in extreme environments [29]. The whole-genome comparison showed that Q1 has extensive similarity to the genomes of other members of the *B*. *cereus* group and the greatest similarity to nonpathogenic strain *B*. *cereus* ATCC 10987. Genomic analysis revealed that *B*. *cereus* Q1 contains several genes related to niche-specific adaptations. [29]. The second largest cluster of strains, ST1295, was grouped near a smaller cluster with a designation of ST90, which had been isolated from liquid eyeliner samples. There were nine strains that were designated ST1295. We found several differences in the two major STs when comparing them to each other. ST1295 had one additional virulence gene, cytK2, that was absent from ST266 and one additional AMR gene, (Gly) *VanZF-Pp*. In the carbohydrate utilization studies, we found that there were four isolates with ST1295 designation that were positive for both esculin and esculin hydrolysis. In ST266, only one isolate was positive for the utilization of both carbohydrates.

We analyzed enterotoxin gene prevalence and distribution. A genotyping strategy confirmed the presence of enterotoxin genes. Virulence/enterotoxin gene patterns have been compiled for *B. cereus,* which has been mainly isolated from foods but also from clinical, soil, and environmental samples worldwide [30]. One study reported that the food matrix might influence the virulence expression of *B. cereus* [31]. In some studies, a connection has been established between toxin gene patterns and the geographical location of isolates. [30]. A more recent study reported strains possessing nhe, hblA, and cytK were predominant in regions with a hot arid climate and only comparable rare in cold continental climates. [32] The most prevalent toxin genes isolated from Chinese food were the *entFM* and the *nheBAC* genes [30]. The most prevalent profile was found in the baby wipes (*nheBAC* and *entFM*), profile #5, compared to the profile seen with dietary supplements and medicated fish food in a study conducted by Carter et al. [5]. Four strains under profile #3 (positive for all the toxin genes except cytK) correlated with the profile reported for powdered infant formula (PIF), also in a study by Carter et al. [5]. Cytotoxin K genes have been found in numerous strains of *B. cereus* and other members of the *B. cereus* group; however, this feature is strain rather than species-specific. [33]. These findings hold true for all of the other virulence genes as well. The *cytK* toxin gene was found to be present in 11 of our strains tested by the PCR assay method (Table 2).

As part of our strategy to better understand the persistence of this organism, we incorporated BTyper as a tool for bioinformatics analysis. BTyper is a genomic computational tool for the analysis of *B. cereus* [12]. The BTyper pipeline has now been incorporated into the CFSAN GalaxyTrakr platform (https://galaxytrakr.org/root/login?redirect=%2F; accessed on 6 May 2022). This computational tool enabled us to understand better how these strains found in this niche could be characterized by identifying virulence genes, assigning *panC* Clade designations, and determining the antimicrobial resistance genes present in each isolate. The virulence genes identified in each strain corresponded with the assigned ST (Figure 2).

*B. cereus* ST1295 was found in two of the strains described as Pure (P) produced by Manufacturer A and three strains from the same manufacturer but identified as Gentle Cleansing (G). Manufacturer B had a total of four *B. cereus* ST1295 strains, two each from Scented (S) and Unscented (U) products. The single strain, ST2103, was found in baby wipes described as Pure (P) from Manufacture A. We examined the ST phylogenetic relatedness of our cosmetics strains with our baby wipe strains. We found most of the cosmetics eyeliner strains belonged to ST90 (Figure 1) and clustered between the two major baby wipe strains, ST266 and ST1295. There was one *Bacillus* reference strain, FRI-35, that belonged to ST90. There was also one cosmetics eyeliner strain that belonged to ST1985. Two strains from cosmetics eye shadows and one ST ND (not determined) appear to be closely related according to the alignment displayed in the phylogenetic tree. One strain from cosmetics eye shadow, ST1317, clustered with reference strain HBL-AI ST1436, a *Bacillus* strain with biotransformation capabilities. We did not find, however, a correlation between the ST, the wipe formula, and geographical origin. Strains of both major STs (ST266 and ST1295) were associated with products from both manufacturers.

The ability of an organism to adapt and survive in various environments is dependent on an assortment of factors, one being the use of carbohydrates as viable resources. To this end, we initiated a study to analyze the carbohydrate utilization of these strains, as recent evidence suggests there is a link between carbohydrate utilization and microbial contamination [18]. Cell surface carbohydrates play a pivotal role in various bacterial functions and activities, including providing defense mechanisms to guard against unfavorable environmental conditions [34]. Carbohydrates, which serve as major energy sources for bacterial growth and metabolic activity, are involved in a variety of cellular processes and are thought to be linked to the evolution of *B. cereus* [35]. Carbohydrates, which serve as major energy sources for bacterial growth and metabolic activity, are involved in a variety of cellular processes and are thought to be linked to the evolution of *B. cereus* [35]. One study described carbohydrate utilization as an important component of maintaining *B. cereus* cell surfaces, such as its capsule and cell wall. Genes for carbohydrate utilization may help to elucidate the molecular basis for bacterial virulence and pathogenicity [34]. The importance of the role carbohydrate utilization plays in the proliferation of *B. cereus* in the environment has only recently been investigated [18].

We decided to analyze the carbohydrate genes found in the *B. cereus* strains recovered from baby wipes in hopes of identifying specific genomic components that facilitate the carbohydrate utilizing abilities and as part of an overall strategy to define a genetic basis of adaptation. The results of the biochemical fermentation assay revealed methyl β-D- glucopyranoside (arbutin), a carbohydrate that utilizes the phosphoenolpyruvate-dependent phosphotransferase system (PTS), was detected in Bc64, Bc186, Bc194, Bc195, Bc198, and Bc201. N-acetyl-glucosamine, D-glucose, trehalose, maltose, and maltotriose are compounds known to support the growth of *B. cereus* [18]. The Cellobiose phosphotransferase system YdjC-like protein gene involved in carbohydrate metabolism was found in ST2103 Bc57 and all ST1295 strains except strain Bc201. In contrast, this gene was only present in three ST266 strains. Clearly, more investigative work should be undertaken to learn about the genetic factors that influence the environmental adaptability of this organism. A part of the overall characterization was to determine if there was an intricate link between the genomic analysis of *B. cereus* recovered from baby wipes and carbohydrate utilization/transport systems identified among these strains.

At the conclusion of our investigation and the carbohydrate studies, we could not find a direct correlation between the presence/absence of certain carbohydrate genes found in these baby wipes strains and the other molecular and biochemical findings. Our investigations revealed that *B. cereus* strains recovered from baby wipe sheets of the same brand can be diverse, yet *B. cereus* strains obtained from different countries may share a similarity in some genetic information and biochemical functions. However, we did find a difference in enterotoxin gene profiles from the same manufacturer within the same country.

Our data present a multifaceted approach characterizing *B. cereus* strains recovered from baby wipes with the application of WGS, biochemical tests, and bioinformatic tools. The utility of a comprehensive approach to helping to differentiate factors that may play a significant role in microbe survival in this environmental niche is essential to our understanding of this organism.

It is critical to note that studies characterizing *B. cereus* strains obtained from several unique sample sources are increasing, and future surveillance studies are warranted to understand the extent of the global prevalence of organisms, especially those found associated with the “built environment.” Future predictions of where an organism can persist and elucidation of its mechanisms of survival remains a complicated story that will require improved detection methods, WGS, and microbiological metadata and potentially contribute to the development of prevention strategies to control this organism’s survival in unwanted environments where it could prove to be harmful to public safety.

## Figures and Tables

**Table 1:** Isolates from baby wipes and cosmetics, manufacturer source, product information, isolation date, and corresponding metadata of strains used in this study.

Isolates ID	Source	Product Type/Brand	Date of Isolation	Country/Origin	Number of Isolates
subA-J	Manufacturer A	Baby wipes P (Pure)	August 2015	Europe	10
subK-T	Manufacturer A	Baby wipes G (Gentle Cleansing)	August 2015	Europe	10
subU-DD	Manufacturer B	Baby wipes S (Scented)	August 2015	Asia	10
subEE-NN	Manufacturer B	Baby wipes U (Unscented)	August 2015	Asia	10
subO2-R2	Manufacturer C	Eyeshadow	November 2015	America	4
subS2-V2	Manufacturer D	Liquid Eyeliner	November 2015	Asia	4

Note that subW is one additional cosmetics isolate that was removed from the table due to the lack of sufficient metadata.

**Table 2:** Enterotoxin gene profiles of *B. cereus* strains isolated from baby wipes as identified using PCR.

Isolate Name	Assembly Name	Target Gene								Profile
		*hblD*	*hblA*	*hblC*	*nheB*	*nheA*	*nheC*	*cytK*	*entFM*	
subA	Bc54	−	−	−	+	−	−	+	+	P-1
subB	Bc55	−	−	−	+	−	−	+	+	P-1
subC	Bc56	−	−	−	+	−	−	−	+	P-2
subD	Bc57	+	+	+	+	+	+	−	+	P-3
subE	Bc58	−	−	−	+	−	−	+	+	P-1
subF	Bc59	−	−	−	+	−	−	−	+	P-2
subG	Bc60	+	+	+	+	+	+	−	+	P-3
subH	Bc61	−	−	−	+	−	−	−	+	P-2
subI	Bc62	−	−	−	+	−	−	−	+	P-2
subJ	Bc63	+	+	+	+	+	+	−	+	P-3
subK	Bc64	−	−	−	+	+	+	+	+	P-4
subL	Bc65	−	−	−	+	−	−	−	+	P-2
subM	Bc177	−	−	−	+	+	+	+	+	P-4
subN	Bc178	−	−	−	+	+	+	−	+	P-5
subO	Bc179	−	−	−	+	+	+	−	+	P-5
subP	Bc180	−	−	−	+	+	+	−	+	P-5
subQ	Bc181	−	−	−	+	+	+	−	+	P-5
subR	Bc182	−	−	−	+	+	+	−	+	P-5
subS	Bc183	−	−	−	+	+	+	−	+	P-5
subT	Bc184	−	−	−	+	+	+	+	+	P-4
subU	Bc185	−	−	−	+	+	+	−	+	P-5
subV	Bc186	−	−	−	+	+	+	−	+	P-5
subW	Bc187	−	−	−	+	+	+	+	+	P-4
subX	Bc188	−	−	−	+	+	+	−	+	P-5
subY	Bc189	−	−	−	+	+	+	−	+	P-5
subZ	Bc190	−	−	−	+	+	+	+	+	P-4
subAA	Bc191	−	−	−	+	+	+	−	+	P-5
subBB	Bc192	−	−	−	+	+	+	−	+	P-5
subCC	Bc193	−	−	−	+	+	+	−	+	P-5
subDD	Bc194	+	+	+	+	+	+	−	+	P-3
subEE	Bc195	−	−	−	+	+	+	−	+	P-5
subFF	Bc196	−	−	−	+	+	+	−	+	P-5
subGG	Bc197	−	−	−	+	+	+	−	+	P-5
subHH	Bc198	−	−	−	+	+	+	−	+	P-5
subII	Bc199	−	−	−	+	+	+	−	+	P-5
subJJ	Bc200	−	−	−	+	+	+	+	+	P-4
subKK	Bc201	−	−	−	+	+	+	−	+	P-5
subLL	Bc202	−	−	−	+	+	+	+	+	P-4
subMM	Bc210	−	−	−	+	+	+	−	+	P-5
subNN	Bc204	−	−	−	+	−	+	+	+	P-6

(+) = positive by PCR; (−) = negative by PCR.

**Table 3:** WGS assembly, genomic information, and GenBank accession numbers of *B. cereus* isolates used in this study.

Isolate ID	Strain	NCBI Accession Number ^†^	BioSample ^†^	Number of CDS ^a^	Gene	ST ^b^
subB	MOD1_Bc55	MIFH00000000	SAMN05608072	5000	5354	1295
subC	MOD1_Bc56	NHTX00000000	SAMN07163330	5279	5611	266
subD	MOD1_Bc57	NHTY00000000	SAMN07163329	5657	6056	2103
subE	MOD1_Bc58	NHTZ00000000	SAMN07163328	5261	5620	266
subF	MOD1_Bc59	NHUA00000000	SAMN07163327	5293	5628	266
subG	MOD1_Bc60	NHUB00000000	SAMN07163326	5299	5598	266
subH	MOD1_Bc61	NHUC00000000	SAMN07163325	5316	5611	266
subI	MOD1_Bc62	NHUD00000000	SAMN07163324	5235	5534	266
subJ	MOD1_Bc63	NHUE00000000	SAMN07163323	5301	5597	266
subK	MOD1_Bc64	NHUF01000000	SAMN07163322	5041	5392	1295
subL	MOD1_Bc65	NHUG00000000	SAMN07163321	5292	5581	266
subM	MOD1_Bc177	NHUH00000000	SAMN07163320	5043	5377	1295
subN	MOD1_Bc178	NHUI00000000	SAMN07163319	5294	5598	266
subO	MOD1_Bc179	NHUJ00000000	SAMN07163318	5310	5622	266
subP	MOD1_Bc180	NHUK00000000	SAMN07163317	5305	5617	266
subQ	MOD1_Bc181	NHUL00000000	SAMN07163316	5227	5551	266
subR	MOD1_Bc182	NHUM00000000	SAMN07163293	5303	5615	266
subS	MOD1_Bc183	NHUN00000000	SAMN07163292	5305	5597	266
subT	MOD1_Bc184	NHUO00000000	SAMN07163291	5075	5409	266
subU	MOD1_Bc185	NHUP00000000	SAMN07163290	5231	5560	266
subV	MOD1_Bc186	NHUQ00000000	SAMN07163277	5305	5631	266
subW	MOD1_Bc187	NHUR00000000	SAMN07163276	5057	5405	1295
subX	MOD1_Bc188	NHUS00000000	SAMN07163275	5299	5609	266
subY	MOD1_Bc189	NHUT00000000	SAMN07163274	5303	5622	266
subZ	MOD1_Bc190	NHUU00000000	SAMN07163273	5060	5368	1295
subAA	MOD1_Bc191	NHUV00000000	SAMN07163272	5317	5615	266
subBB	MOD1_Bc192	NHUW00000000	SAMN07163271	5305	5599	266
subCC	MOD1_Bc193	NHUX00000000	SAMN07163270	5291	5598	266
subDD	MOD1_Bc194	NINF00000000	SAMN07163269	5215	5541	266
subEE	MOD1_Bc195	NHUY00000000	SAMN07163268	5303	5613	266
subFF	MOD1_Bc196	NING00000000	SAMN07163267	5304	5624	266
subGG	MOD1_Bc197	NINH00000000	SAMN07163266	5290	5619	266
subHH	MOD1_Bc198	NHUZ00000000	SAMN07163265	5301	5589	266
subII	MOD1_Bc199	NHVA00000000	SAMN07163264	5298	5590	266
subJJ	MOD1_Bc200	NHVB00000000	SAMN07163263	5297	5596	266
subKK	MOD1_Bc201	NHVC00000000	SAMN07163262	5060	5380	1295
subLL	MOD1_Bc202	NHVD00000000	SAMN07163261	5234	5522	266
subMM	MOD1_Bc210	NHVE00000000	SAMN07163260	5296	5592	1295
subNN	MOD1_Bc204	NHVF00000000	SAMN07163259	5058	5383	266

^†^ denotes NCBI GenBank accession number and Bionumber; ^a^ Number of CDS represent CDS Coding DNA Sequences and ^b^ denotes Sequence Types (ST), MLST. subA was inadvertently not submitted to NCBI.

**Table 4:** Carbohydrate utilization-related genes that are present in *B. cereus* strain (Bc57) ST 2103.

Carbohydrate Gene	Molecular/Enzyme Function
Transcriptional activator of acetoin dehydrogenase operon AcoR	ATP binding ^a^
Sucrose-6-phosphate hydrolase (EC 3.2.1.26) B	Metabolism of disaccharides ^b^, Galactose metabolism, Starch, and sucrose metabolism ^c^
Glycerate_kinase_ (EC 2.7.1.31)	Glycolate and glyoxylate degradation ^b^, Biosynthesis of antibiotics ^c^
sucrose operon repressor ScrR, LacI family	DNA Binding ^a^
Fructokinase (EC 2.7.1.4)	Metabolism of disaccharides ^b^, Amino sugar, and nucleotide sugar metabolism ^c^
1-phosphofructokinase (EC 2.7.1.56)	Degradation of hexoses ^b^, Fructose, and mannose metabolism ^c^
2-hydroxy-3-oxopropionate reductase (EC 1.1.1.6A)	Degradation of sugar acids ^b^, Glyoxylate and dicarboxylate metabolism ^c^
3-hydroxybutyrate dehydrogenase (EC 1.1.1.3A)	Butanoate metabolism ^c^
Phosphoglycolate phosphatase (EC 3.1.3.18)	Degradation of pentoses ^b^, Biosynthesis of antibiotics ^c^
Possible glyoxylase family protein (Lactoylglutathione lyase) (EC 4.4.1.5)	Methylglyoxal degradation ^b^, Pyruvate metabolism ^c^
Lactoylglutathione lyase	A methylglyoxal degradation ^b^, Pyruvate metabolism ^c^
Dihydrolipoamide dehydrogenase of acetoin dehydrogenase (EC 1.8.1.4)	Acetyl CoA biosynthesis ^b^ Glycolysis/Gluconeogenesis ^c^
Dihydrolipoamide acetyltransferase component (E2) of acetoin dehydrogenase complex (EC 2.3.1.12)	Acetyl CoA biosynthesis ^b^, Glycolysis/Gluconeogenesis ^c^
D-alanine aminotransferase (EC 2.6.1.21)	Alanine metabolism ^b^, D-alanine ^c^
Aldehyde dehydrogenase (EC 1.2.1.3) in 4-hydroxyproline_catabolic_gene_cluster	Alanine metabolism ^b^, Glycolysis/Gluconeogenesis, Carbohydrate metabolism ^c^
D-glycero-beta-D-manno-heptose-1,7-bisphosphate 7-phosphatase (EC 3.1.3.82); possible Histidinol-phosphatase (EC 3.1.3.15) CDS	Lipopolysaccharide biosynthesis ^c^

SOURCES of information: ^a^ UniProKB-KW Ref *Bacillus Subtilis*, ^b^ Brenda enzyme database, ^c^ KEGG a genome annotation database.

## Data Availability

Data supporting the findings of this study are contained within the manuscript.

## Supplementary Materials

The following supporting information can be downloaded at: https://www.mdpi.com/article/10.3390/microorganisms10091779/s1, Supplemental Table S1. PCR primers used for multiplex PCR detection of enterotoxin genes in *B. cereus* isolates. Supplemental Table S2. Biochemical carbohydrate fermentation test. Supplemental Table S3. Pearson Analysis. Supplemental Table S4. The presence/ absence of all nonredundant carbohydrate genes found in baby wipe isolates as determined by BLAST analysis.
