# Supplementary material for: Characterization and Genetic Diversity of Bacillus cereus Strains Isolated from Baby Wipes
Source: Microorganisms. 2022 Sep 3;10(9):1779. doi: 10.3390/microorganisms10091779 (PMC9502454; doi:10.3390/microorganisms10091779)
Supplement: Supplementary file 1 [file microorganisms-10-01779-s001.zip › Supplemental Table S1.pdf]

Supplemental Table S1. PCR primers used for multiplex PCR detection of enterotoxin genes in *B. cereus* isolates

Target gene	Primer[a]	Primer sequence (5′–3′)	Primer size (bp)	T_m (°C)	Product size (bp)	Primer concentration (μM)	Reference
hblC	FHblC	CCTATCAATACTCTCGCAA	19	54	695	0.4	Ngamwongsatit, 2008
	RHblC	TTTCCTTTGTTATACGCTGC	20	56			
hblD	FHD	GAAACAGGGTCTCATATTCT	20	56	1018	0.4	Thaenthanee, 2004
	RHD2	CTGCATCTTTATGAATATCA	20	52			
hblA	FHblA	GCAAAATCTATGAATGCCTA	20	54	884	0.4	Ngamwongsatit, 2008
	RHblA	GCATCTGTTCGTAATGTTTT	20	54			
nheA	F2NheA	TAAGGAGGGGCAAACAGAAG	20	60	759	0.2	Ngamwongsatit, 2008
	RNheA	TGAATGCGAAGAGCTGCTTC	20	60			
nheB	F2NheB	CAAGCTCCAGTTCATGCGG	19	60	935	0.2	Ngamwongsatit, 2008T
	RNheB	GATCCCATTGTGTACCATTG	20	58			
nheC	FNheC	ACATCCTTTTGCAGCAGAAC	20	58	618	0.2	Ngamwongsatit, 2008
	R2NheC	CCACCAGCAATGACCATATC	20	60			
cytK	FCytK	CGACGTCACAAGTTGTAACA	20	58	565	0.2	Ngamwongsatit, 2008
	R2CytK	CGTGTGTAAATACCCCAGTT	20	58			
entFM	FEntFM	GTTCGTTCAGGTGCTGGTAC	20	62	486	0.2	Ngamwongsatit, 2008
	REntFM	AGCTGGGCCTGTACGTACTT	20	62			

[a] F = forward primer; R = reverse primer.
