# Supplementary material for: Characterization and Genetic Diversity of Bacillus cereus Strains Isolated from Baby Wipes
Source: Microorganisms. 2022 Sep 3;10(9):1779. doi: 10.3390/microorganisms10091779 (PMC9502454; doi:10.3390/microorganisms10091779)
Supplement: Supplementary file 1 [file microorganisms-10-01779-s001.zip › Supplemental Table S2.pdf]

Supplemental Table S2. Biochemical carbohydrate fermentation test.

ISOLATES	MOD1	CARBOHYDRATE D-Ribose	DAY (+)	Methyl α-D-glucopyroside (unit of Cyclodextrin	DAY (+)	Methyl β- D-glucopyroside (Arbutin)	DAY (+)	Cyclodextrin	DAY (+)	N-Acetyl-D-glucosamine	DAY(+)
**SubA**	**MOD1_Bc54**	**(+)**	**1-7 (+)**	**(-)**		**(-)**		**(+)**	**4**	**(+)**	**1-7 (+)**
**SubB**	**MOD1_Bc55**	**(+)**		**(-)**		**(-)**		**(+)**	**5**	**(+)**	
**SubC**	**MOD1_Bc56**	**(+)**		**(-)**		**(-)**		**(+)**	**5**	**(+)**	
**SubD**	**MOD_Bc57**	**(+)**		**(-)**		**(-)**		**(+)**	**7***	**(+)**	
**SubE**	**MOD1_Bc58**	**(+)**		**(-)**		**(-)**		**(+)**	**5**	**(+)**	
**SubF**	**MOD1_Bc59**	**(+)**		**(-)**		**(-)**		**(+)**	**4**	**(+)**	
**SubG**	**MOD1_Bc60**	**(+)**		**(-)**		**(-)**		**(+)**	**4**	**(+)**	
**SubH**	**MOD1_Bc61**	**(+)**		**(-)**		**(-)**		**(+)**	**4**	**(+)**	
**SubI**	**MOD1_Bc62**	**(+)**		**(-)**		**(-)**		**(+)**	**5**	**(+)**	
**SubJ**	**MOD1_Bc63**	**(+)**		**(-)**		**(-)**		**(+)**	**5**	**(+)**	
**SubK**	**MOD1_Bc64**	**(+)**		**(-)**		**(+)**	**3**	**(+)**	**4**	**(+)**	
**SubL**	**MOD1_Bc65**	**(+)**		**(-)**		**(-)**		**(+)**	**4**	**(+)**	
**SubM**	**MOD1_Bc177**	**(+)**		**(-)**		**(-)**		**(+)**	**5**	**(+)**	
**SubN**	**MOD1_Bc178**	**(+)**		**(-)**		**(-)**		**(+)**	**4**	**(+)**	
**SubO**	**MOD1_Bc179**	**(+)**		**(-)**		**(-)**		**(+)**	**7***	**(+)**	
**SubP**	**MOD1_Bc180**	**(+)**		**(-)**		**(-)**		**(+)**	**5**	**(+)**	
**SubQ**	**MOD1_Bc181**	**(+)**		**(-)**		**(-)**		**(+)**	**5**	**(+)**	
**SubR**	**MOD1_Bc182**	**(+)**		**(-)**		**(-)**		**(+)**	**5**	**(+)**	
**SubS**	**MOD_Bc183**	**(+)**		**(-)**		**(-)**		**(+)**	**4**	**(+)**	
**SubT**	**MOD1_Bc184**	**(+)**		**(-)**		**(-)**		**(+)**	**4**	**(+)**	

**SubU**	**MOD1_Bc185**	**(+)**	**(-)**	**(-)**		**(+)**	**4**	**(+)**
**SubV**	**MOD1_Bc186**	**(+)**	**(-)**	**(+)**	**2**	**(+)**	**4**	**(+)**
**SubW**	**MOD1_Bc187**	**(+)**	**(-)**	**(-)**		**(+)**	**4**	**(+)**
**SubX**	**MOD1_Bc188**	**(+)**	**(-)**	**(-)**		**(+)**	**4**	**(+)**
**SubY**	**MOD1_Bc189**	**(+)**	**(-)**	**(-)**		**(+)**	**4**	**(+)**
**SubZ**	**MOD1_Bc190**	**(+)**	**(-)**	**(-)**		**(+)**	**5**	**(+)**
**SubAA**	**MOD1_Bc191**	**(+)**	**(-)**	**(-)**		**(+)**	**4**	**(+)**
**SubBB**	**MOD1_Bc192**	**(+)**	**(-)**	**(-)**		**(+)**	**4**	**(+)**
**SubCC**	**MOD1_Bc193**	**(+)**	**(-)**	**(-)**		**(+)**	**4**	**(+)**
**SubDD**	**MOD1_Bc194**	**(+)**	**(-)**	**(+)**	**4**	**(+)**	**5**	**(+)**
**SubEE**	**MOD1_Bc195**	**(+)**	**(-)**	**(+)**	**3**	**(+)**	**5**	**(+)**
**SubFF**	**MOD1_Bc196**	**(+)**	**(-)**	**(-)**		**(+)**	**5**	**(+)**
**SubGG**	**MOD1_Bc197**	**(+)**	**(-)**	**(-)**		**(+)**	**5**	**(+)**
**SubHH**	**MOD1_Bc198**	**(+)**	**(-)**	**(+)**	**3**	**(+)**	**4**	**(+)**
**SubII**	**MOD1_Bc199**	**(+)**	**(-)**	**(-)**		**(+)**	**5**	**(+)**
**SubJJ**	**MOD1_Bc200**	**(+)**	**(-)**	**(-)**		**(+)**	**5**	**(+)**
**SubKK**	**MOD1_Bc201**	**(+)**	**(-)**	**(+)**	**3**	**(+)**	**5**	**(+)**
**SubLL**	**MOD1_Bc202**	**(+)**	**(-)**	**(-)**		**(+)**	**5**	**(+)**
**SubMM**	**MOD1_Bc210**	**(+)**	**(-)**	**(-)**		**(+)**	**5**	**(+)**
**SubNN**	**MOD1_Bc204**	**(+)**	**(-)**	**(-)**		**(+)**	**5**	**(+)**
**(+) Control**	**14579**	**(+)**						
**(-) Control**	**14579**	**(-)**						

*Phenotypic results of carbohydrate utilization after 7 consecutive days indicated by a color change from red to yellow in the media.
